# ZipAEr: A compressive convolutional autoencoder for high-dimensional spatial omics data at subcellular resolution

**DOI:** 10.21203/rs.3.rs-7541289/v1

**Published:** 2025-09-17

**Authors:** Shiva Kazempour, Javad Razavian, Sogand Sajedi, Miranda E. Orr, Megan S. Pater, Grant R. Kolar, Soroosh Solhjoo, Habil Zare

**Affiliations:** 1 Department of Cell Systems & Anatomy, The University of Texas Health Science Center, San Antonio, Texas, USA; 2 Glenn Biggs Institute for Alzheimer’s & Neurodegenerative Diseases, San Antonio, Texas, USA; 3 Department of Neurology, Washington University School of Medicine, St. Louis, MO, USA; 4 US Department of Veterans Affairs, Washington, WA, USA; 5 Saint Louis University, St. Louis, MO, USA; 6 Department of Biological Sciences, Missouri University of Science and Technology, Rolla, MO, USA; 7 Johns Hopkins University School of Medicine, Baltimore, Maryland, USA; 8 F. Edward Hébert School of Medicine, Bethesda, Maryland, USA

## Abstract

Recent advances in spatial transcriptomics have produced rich, high-throughput datasets, but their biological interpretation remains challenging due to analytical complexity. We present ZipAEr, a convolutional autoencoder tailored to extract informative latent features from spatial omics data. Unlike traditional methods that reduce data at the cell level, ZipAEr operates at the transcript level, preserving both subcellular and extracellular spatial context. Conventional autoencoders, built for images with three channels (red, green, blue), cannot handle spatial omics data with thousands of input channels representing genes and proteins. ZipAEr addresses this by reducing both spatial dimensions and channel count through its convolutional layers. It also introduces channel weighting in the loss function to ensure balanced representation of lowly expressed genes. ZipAEr effectively compresses spatial omics data by two to three orders of magnitude while preserving key spatial and molecular features. The resulting latent representation enables downstream analyses, such as classification and clustering, which would otherwise be computationally infeasible with raw data.

## Introduction

Traditional transcriptomic methods, such as single-cell RNA sequencing (scRNA-seq), provide high-throughput gene expression data^1^ but lack spatial coordinates of cells and transcripts due to cell dissociation. The lack of spatial data makes it challenging to study the interactions of cells with their microenvironment, and the effects of pathological factors^2^, such as tumors^3,4^, neurofibrillary tangles, or amyloid plaques on surrounding cells^5,6^. In contrast, modern spatial omics technologies facilitate the profiling of gene expression while retaining the spatial location of each RNA transcript within intact tissue. Some technologies simultaneously detect hundreds of proteins and measure their spatial distributions within the same tissue^7–11^.

The CosMx Spatial Molecular Imager is an *in situ* sequencing technology with a relatively high spatial resolution. It provides multiplexed, image-based spatial transcriptomics by detecting and quantifying more than 6,000 RNA transcripts at subcellular resolution (20–50nm)^12^. CosMx is particularly valuable for studying complex tissue, such as the brain, where cellular heterogeneity and spatial distribution of gene expression are significant factors in function and disease.

Computational methods for analyzing spatial omics data have traditionally focused on cell-level representations. These approaches often utilize clustering^13^, spatial correlation, and graph-based models^14,15^ to infer cell-cell interactions^16^. For example, BayesSpace applies a Bayesian model that leverages spatial neighborhoods to achieve subspot resolution clustering on low-resolution data^17^. SpaGCN integrates spatial location, gene expression, and histological features using a graph convolutional network to uncover spatial domains^18^. More recently, GraphST introduced a graph-based contrastive^19^ self-supervised learning framework that enables spatial clustering, multi-sample integration, and spatially informed cell-type deconvolution^20^ with high fidelity in capturing tissue architecture^21^.

Cellular-level approaches to analyzing spatial omics data have several limitations. While clustering is a commonly used technique for cell-level spatial data analysis, it often lacks reproducibility due to the stochastic nature of clustering algorithms^22^. Moreover, clustering assumes discrete groups of cells, while cellular state is often a continuum, particularly in contexts such as differentiation and tumor progression^23^. Another challenge in spatial data analysis at the cellular level is identifying cell boundaries accurately, especially in brain tissue, which contains cells with complex shapes, such as neurons^24^ and astrocytes^25^. Furthermore, cell-level analysis methods are inherently limited in their ability to capture transcriptomic and proteomic variations at subcellular resolution. Such variations can be critical for understanding the biology of diseases. For example, a prominent pathological feature of Alzheimer’s disease is tau mislocalization to neuronal somata and dendrites^26–28^, which may not be detectable with cell-level analyses.

To analyze the spatial omics data at the subcellular level, we considered autoencoders. Autoencoders are neural networks^29^ designed to reduce the dimensionality of the data^30,31^ while preserving their essential features^32^. An autoencoder comprises two components: an encoder, which encodes raw data from the input space into a significantly smaller latent space^33^, and a decoder, which converts the latent representation back to the input space. An autoencoder is successfully trained if the decoder’s output is a high-fidelity reconstruction of the original input to the encoder, with some acceptable loss, suggesting that the essential features of the input are compressed in the smaller latent representation. Conventional autoencoders, originally designed to analyze natural images with three color channels, cannot directly scale to spatial omics data with thousands of input channels.

Each autoencoder architecture is optimized to solve a specific problem, ranging from data compression and denoising to anomaly detection and generative modeling^34^. For example, vanilla autoencoders are used for dimensionality reduction and feature learning^35,36^, and denoising autoencoders are designed to remove noise from data^37^. Sparse autoencoders^38^ enforce sparsity to learn meaningful representations, making them useful for feature extraction^39^ and anomaly detection^40^. Contractive autoencoders^41^ use regularization to learn robust representations, especially in noisy environments^42^. For generative tasks, variational autoencoders (VAE)^33,43^ model probabilistic distributions to generate new data samples, whereas adversarial autoencoders (AAE)^44^ combine autoencoding with Generative Adversarial Network (GANs)^45^ to create realistic synthetic data. Convolutional autoencoders^46^ utilize convolutional layers to reduce image size while increasing the number of channels^47^. They have also been used for image super-resolution^48^.

Autoencoders have demonstrated remarkable performance in analyzing omics data^49–51^. Data compression and noise reduction, which are crucial for analyzing single-cell omics data, are effectively handled with autoencoders^52–55^. Additionally, autoencoders can facilitate the integration of different omics data types, including gene expression, miRNA expression, and DNA methylation^56,57^. Denoising autoencoders have been used to create robust representations of multi-omics data, which can then be applied to cancer prognostication^58^. Variational autoencoders and their variants have been used for cancer subtype detection by extracting low-dimensional representations that capture omics dependencies^59–61^. Some other autoencoder variants have been employed for clustering patients into biologically meaningful subgroups based on multi-omics data^62,63^.

Here, we introduce ZipAEr, a novel deep learning framework for spatial transcriptomics analysis at the subcellular level. Designed based on the convolutional autoencoder architecture, ZipAEr extracts meaningful latent representations directly from CosMx transcriptomic data. By capturing transcriptomic variations at subcellular resolution, ZipAEr facilitates the characterization of cell states and microenvironment interactions.

## Methods and Results

### Data

Spatial transcriptomic data acquired using the CosMx Spatial Molecular Imager (NanoString) from one human frontal cortex tissue slice. We downloaded this dataset from the NanoString website^64^ and used it to train and test our models. A CosMx image can be considered a multi-channel tensor where the spatial dimensions correspond to the pixel coordinates. Each of the 6078 channels has a binary value indicating the presence or absence of a particular gene transcript in the corresponding pixel. The resolution of each field of view^65^ (FOV) was 4256 × 4256 pixels, where each pixel was a 20nm ×20nm square. The downloaded dataset consisted of 392 FOVs, collectively forming a 7,100-megapixel image with 6078 channels. Our approach also applies to spatial transcriptomic data generated using similar spatial omics technologies.

### Preprocessing

To keep the spatial context while maintaining computational feasibility, we divided each FOV into smaller square regions called “patches” centered around each cell as explained below. A custom PyTorch Dataset class, CosMxDataset, constructed the patches and allowed for batched processing of the spatial transcriptomic data via PyTorch DataLoader.

#### Extracting data patches

Each FOV was systematically divided into patches based on cell coordinates, ensuring each cell was at the center of exactly one patch. The dataset, therefore, contained as many patches as there were cells. Each patch was large enough to include a few neighboring cells, ensuring every transcript included in a patch was analyzed along with other transcripts of the corresponding cell and its immediate microenvironment. The patch extraction process included the following steps:

1. Identify the coordinates of the center of each cell.
2. Extract a 600 × 600 pixel square patch centered around each cell.
3. Construct a tensor representation of each patch, where the genes serve as separate channels.

#### Patch overlap

Although each patch was uniquely assigned to a central cell, patches corresponding to neighboring cells could overlap. Due to this redundancy, transcripts at the edges of a given patch might also be included in overlapping patches, thereby preserving contextual information. The amount of overlap depended on the patch size and the density of the cells. By incorporating overlapping patches, the model received additional context for learning spatial transcriptomic patterns.

#### The reduction parameter

Given the large number of channels (more than 6000) and the high resolution of the CosMx data, keeping patches in full resolution would be computationally expensive. To optimize memory usage, we introduced a reduction parameter *r* to decrease the spatial resolution of each patch, enabling the model to operate on the large CosMx data without exceeding GPU memory limits.

Reduction parameter *r* = 2 reduced the number of pixels and the memory usage by a factor of *r*^2^ = 4, achieving a balance between computational cost and spatial resolution. Preprocessing resulted in 190K patches of size 300 × 300 pixels.

### Model

**Figure 1.**
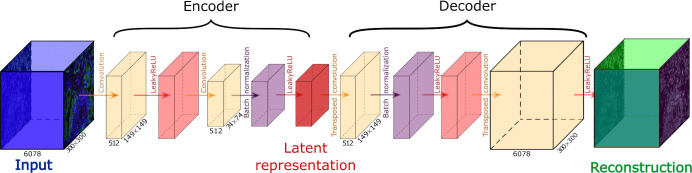
The schematic view of the ZipAEr2, a compressive convolutional autoencoder with 2 convolutional layers. Boxes represent tensors, and each arrow represents a computational layer. The decoder mirrors the encoder’s structure, using transposed convolutions. For brevity, tensor shapes are omitted when they remain unchanged from the previous layer. The input is a tensor of size 300 × 300 with 6078 channels (i.e., genes), which is compressed using convolutional layers (orange arrows) in the encoder. Unlike conventional autoencoders, both the spatial dimensions and depth of the input tensor are reduced. LeakyReLU (red arrows) is used as the activation function. Batch normalization (purple arrows) is applied after the last convolution in the encoder and after the first convolution in the decoder. The encoder produces a 512 × 74 × 74 latent representation tensor (red box), the decoder uses to reconstruct (green box) the input.

ZipAEr is a *compressive convolutional autoencoder*, specifically designed to learn compact representations of high-dimensional spatial transcriptomic data. The architecture of our autoencoder is symmetric, with the encoder and decoder having the same number of layers. Unlike traditional autoencoders that typically focus on reducing only the spatial resolution, our model compresses data in a novel way along both the spatial and channel axes (Fig. 1).

We developed and evaluated two models, denoted as ZipAEr2 (Fig. 1) and ZipAEr3, where the trailing digit specifies the number of convolutional layers in the encoder and decoder. In both models, the encoder received an input tensor of shape 6078 × 300 × 300, where 6078 was the number of transcript channels. A series of 3 × 3 convolutional layers with a stride of 2 progressively downsampled the data in the encoder. We did not apply padding^66^ unless explicitly stated below.

In ZipAEr2, the first convolutional layer reduced the spatial resolution from 300 × 300 to 149 × 149 while also reducing the number of channels from 6078 to 512. Then, the LeakyReLU^67^ activation function was applied to facilitate modeling complex nonlinear relationships. The second convolutional layer reduced the spatial resolution from 149 × 149 to 74 × 74, without changing the number of channels, resulting in a 512 × 74 × 74 latent tensor. Batch normalization^68^ was applied after the encoder’s final convolutional layer^69^ to stabilize training, and was followed by the LeakyReLU activation function. The decoder mirrored this structure using transposed convolutions to reconstruct the original spatial dimensions and transcript channels. Specifically, the 512 × 74 × 74 latent tensor was converted to a 512 × 149 × 149 tensor, followed by batch normalization and LeakyReLU. Then, a second transposed convolution with an output padding of 1 and LeakyReLU activation reconstructed a tensor with the original shape of 6078 × 300 × 300.

The deeper ZipAEr3 model extended the described architecture of ZipAEr2 by introducing a third convolutional layer in the encoder, further reducing the spatial resolution from 74 × 74 to 36 × 36 and the number of channels from 512 to 256. Then, the latent tensor was generated using batch normalization and LeakyReLU activation. Accordingly, an extra transposed convolutional layer with an output padding of 1 was included as the first layer of the ZipAEr3 decoder to reshape the tensor from 256 × 36 × 36 back to 512 × 74 × 74. After applying batch normalization and LeakyReLU, the subsequent layers replicated the architecture of the ZipAEr2 decoder.

### Training

We addressed the imbalance in gene expression using a weighted loss function,

(1) $$ℒ=10^{6}\;\underset{i}{\text{mean}}\left(\frac{\text{median}\left(s_{i}\right)}{s_{i}}{\text{MSE}}_{i}\right),$$

where $s_{i}$ denotes the total expression of gene i and ${MSE}_{i}$ its corresponding mean squared error^70^. We normalized the expression levels using their median to avoid extremely small scaling factors. The constant factor of $10^{6}$ was introduced to improve readability in figures and tables.

The model was trained using the Adam optimizer^71,72^, which is widely used due to its adaptive learning rate and momentum-based updates. The optimizer was configured using an initial learning rate of 10^−3^. We used a scheduler to dynamically adjust the learning rate. The scheduler followed a *ReduceLROnPlateau* strategy^73^, where the learning rate was reduced by a factor of 0.3 when the validation loss did not improve for 8 consecutive epochs, using a threshold of 0.01 to define a plateau (i.e., no improvement). Training was performed on 3 compute nodes, each running 3 GPUs (a total of 9 GPUs), using PyTorch Lightning^74^ as the training engine.

**Figure 2.**
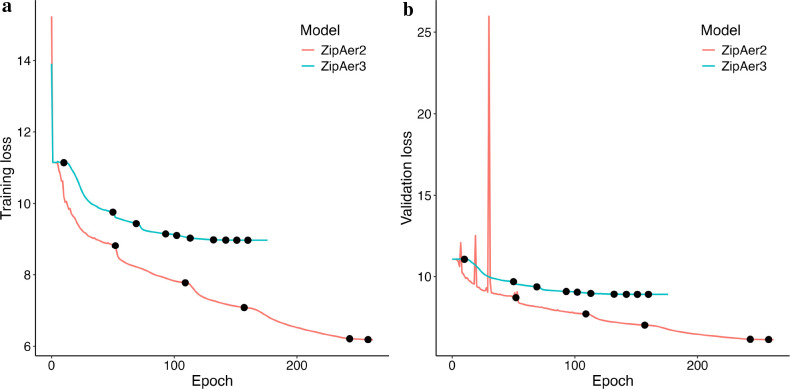
Learning (a) and validation (b) loss for the ZipAEr models. Black dots mark the epochs where the learning rate was reduced by a factor of 0.3, as determined by the learning rate scheduler.

Due to the large size of the spatial transcriptomic data and the 40GB memory limit per GPU, we could not include more than 2 samples (i.e., patches) in a batch. Using 9 GPUs, only 18 samples could be analyzed in one iteration, which was too few for a robust gradient update. As a remedy, we implemented a gradient accumulation strategy^75^ to enable training with an effectively larger batch size. Each of the 9 GPUs processed a batch of 2 samples per iteration, and gradients were accumulated and updated once over 4 iterations. This gradient accumulation strategy resulted in an effective batch size of 72 in one training step. Therefore, 2.6K steps were required to complete 1 epoch of training over all 190K samples. Each model was trained over 500 epochs, and training checkpoints were saved and retrieved as needed to ensure robustness across runs (Fig. 2).

We trained the models using A100^76^ GPUs, each with 40 GB of memory. With a batch size of 2, the memory usage was around 35 GB and was comparable across both models (Table 1). This was expected, as the data volume was reduced by an order of magnitude in the first encoder layer in both models. Additionally, the number of model parameters was negligible compared to the input size, meaning that the input tensor accounted for most of the GPU memory usage.

### Evaluation

**Figure 3.**
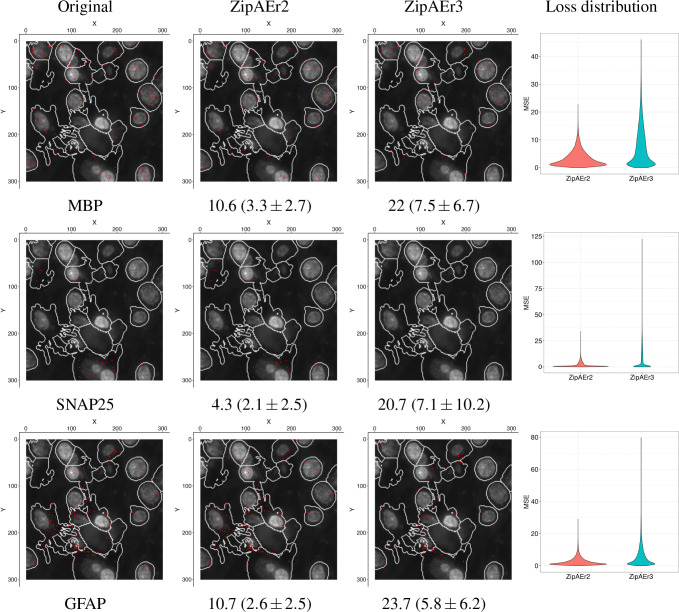
Reconstruction accuracy of ZipAEr models on a randomly selected representative patch of size 12 μm × 12 μm. The cell boundaries and nucleolar content, shown in white as background, were included in the downloaded data and were not a result of our analysis. Red dots indicate transcript locations. Each row corresponds to a specific transcript, indicated under the original expression, which is shown in the first column. The reconstructed outputs from ZipAEr2 and ZipAEr3 models are shown in the second and third columns, respectively. Below each plot, the scaled weighted loss (Equation 1) for the corresponding transcript is listed. The numbers in parentheses represent the mean ± standard deviation of the loss across all validation patches, and the corresponding loss distributions are shown using violin plots in the fourth column. ZipAEr3 misses more transcripts than ZipAEr2 and has larger losses in reconstructing these exemplar genes, which is expected due to ZipAEr2’s smaller global loss (Table 1 and Fig. 2).

Using the latent representation to reconstruct the original input, we qualitatively compared the two variants of the ZipAEr model. Visualization of three neurodegeneration- and cell type-specific transcripts with low, intermediate, and high expression levels indicated that both models achieved acceptable performance for compressing the spatial transcriptomic data in the latent space (Fig. 3).

The ZipAEr3 model exhibited more false positive expression and false negatives than the shallower ZipAEr2 model, which was expected due to its latent representation being an order of magnitude smaller. Thus, for downstream analyses that require accuracy, ZipAEr2 would be more appropriate, whereas ZipAEr3 is better suited for faster analyses on relatively larger datasets. Also, the slight diminishing performance of ZipAEr3 revealed the limits of data compression using a ZipAEr architecture. Particularly, models deeper (with a smaller latent space) than ZipAEr3 are expected to lose a significant amount of information.

#### Applications in molecular biology

The latent features derived from the ZipAEr models provide a compact representation of spatial transcriptomic data that can be useful in answering biological questions. Neurons with neurofibrillary tangles (NFTs)^77^ are hallmark pathogenic features of Alzheimer’s disease. These neurons release pathological tau protein and damage-associated signals into their microenvironments, activating microglia and astrocytes and leading to chronic neuroinflammation^78,79^. This inflammatory response is hypothesized to amplify neuronal damage and synaptic loss, thereby accelerating the progression of Alzheimer’s disease^80–82^. The tau protein is encoded by the microtubule-associated protein tau (*MAPT*) gene^83^. We hypothesized that the influence of a *MAPT*-expressing neuron on its microenvironment is detectable in the transcriptomic profile of its respective patch.

To demonstrate the practical utility of ZipAEr, we trained a 5-layer classifier using latent features extracted from ZipAEr2 and compared its performance to a baseline classifier trained on unprocessed spatial transcriptomic data. Only patches containing a centrally located neuron were used in this experiment. Patches were labeled positive if the neuron expressed *MAPT*, and negative otherwise.

In both classifiers, convolutional layers with 3 × 3 kernels, stride 2, and no padding were used, each followed by a LeakyReLU activation. The input to the 5-layer classifier was a latent tensor of shape 512 × 74 × 74, which was progressively compressed through five convolutional layers into tensors of shape 128 × 36 × 36, 64 × 17 × 17, 32 × 8 × 8, 16 × 3 × 3, and finally 16 × 1 × 1. The resulting tensor was flattened^84,85^, followed by dropout^86^ with a probability of 0.5, which was applied during training only. The final layer was fully connected^84^ with two output values corresponding to the patch label. The architecture of the baseline classifier consisted of the untrained ZipAEr2 encoder followed by the 5-layer classifier. The two classifiers were intentionally designed to be similar in both parameter count and complexity, allowing for a fair comparison. Their difference was that the first classifier leveraged the encoder of our ZipAEr2, which was pretrained^87,88^ using our approach, whereas all parameters had to be learned from scratch in the second (i.e., baseline) classifier.

We trained the classifiers using 70% of the data and tested them on the remaining 30%. The five-layer classifier, which used ZipAEr2’s latent representation, achieved an accuracy of 69%, a recall (sensitivity) of 69%, a precision (positive predictive value) of 69%, and a specificity (true negative rate) of 69%^89^. These results outperformed the baseline classifier, which yielded 70% accuracy, 66% recall, 64% precision, and 60% specificity—underscoring the value of the latent representation obtained from our trained encoder.

### Technical challenges and considerations

The relatively large size of each sample and the high number of channels led to computational challenges, which we addressed using the technologies and approaches described below.

To create a patch, we needed to determine the expression values of more than 6K transcripts for all pixels in the patch. In the CosMx raw data, each transcript is recorded on a single row, including its spatial coordinates, transcript name, coordinates relative to the FOV, the Z-slice image number^90^, and the subcellular compartment (nuclear, membrane, or cytoplasmic) in which the transcript was detected. We needed to inspect all rows of data to determine which transcripts were detected within a patch boundary. Loading the entire 2GB dataset at the beginning of each training would take about 20–30 minutes, which was inefficient. We only needed the x and y coordinates of a transcript to determine whether it belonged to a particular patch. To efficiently read the data, we used the *Apache Parquet*^91^ format, which is columnar, making it more efficient for selective data access compared to row-based formats like CSV. Using the *Apache Parquet* format allowed us to load only the necessary columns (i.e., the transcript names and their x,y coordinates), resulting in significant time and memory savings at the start of each training.

Even when we loaded only the spatial coordinates and transcript names into memory, the resulting object was too big for serial querying by patch, which would drastically slow the patch creation process. We addressed this challenge using *Spark*^92^, which enabled us to utilize all available CPU cores simultaneously and perform simple queries efficiently. We first joined^93^ the large dataset of spatial coordinates and transcript names with the small dataset of patch center coordinates. Then, we filtered the transcripts of each patch simply by checking whether the distance between their spatial coordinates and the patch center was less than the patch size. This led to a sparse representation for each patch as a dataset with these columns: the patch identifier, the transcript identifier, and the x and y spatial coordinates within the patch. We converted this sparse representation to full tensors in the training phase.

To save the intermediate file, we chose the binary Parquet^94^ format, which produces significantly smaller files compared to the more common CSV format. Parquet also enables faster loading and querying, owing to its support for query-pushdown^95^. Another benefit is that column types are explicitly defined within a Parquet file, eliminating the need to infer or manually specify them during loading.

Common parallelization techniques include data parallelization, task parallelization, and hybrid approaches combining both^96,97^. Considering the negligible size of the model compared to the input data in ZipAEr, we opted to use data parallelization in the training phase. Specifically, we used multiple GPUs, each of which had the whole model loaded into its memory and was responsible for computing the forward pass and the corresponding gradient using a very small subset of patches (e.g., 2 patches in our experiments). Then, gradient accumulation^75^ was applied to calculate the gradients based on a larger effective batch size in each step of gradient descent. This technique led to an effective batch size of 72 in our experiments. We used the *PyTorch Lightning*^98^ framework to implement the above approach and leverage multiple GPUs on each machine as well as multiple machines in our SLURM^99^ cluster.

Initially, we attempted to precompute the latent space and store the representations for visualization and other downstream applications, but this approach resulted in too many large files (i.e., 190K files, each with a size of about 5 MB in our experiments). To prevent file system overload, we archived all files into a single tar^100^ file and developed a custom tar-based data loader. Our alternative approach was to compute the encoder’s forward pass on the fly, which was just as fast and efficient as reading the stored latent representations from the file system.

## Discussion

Recent advances in spatial omics^12,101–103^ present both opportunities and challenges: opportunities for exciting biological discoveries^104–106^ through the investigation of subcellular molecular signatures^107–109^ and cell–microenvironment interactions, and challenges in efficiently analyzing the resulting large, high-dimensional data^110–113^.

Here, we introduce ZipAEr, a novel deep learning framework designed to analyze super-high-resolution spatial transcriptomic data. Our approach circumvents the limitations of traditional cell-level aggregation by processing the data in its native, high-dimensional format. By defining “patches” centered around individual cells, we preserve the intricate spatial context of each transcript, including its subcellular location and its immediate microenvironment. This strategy is particularly advantageous for studying complex tissues like the brain, where cell segmentation is notoriously difficult and subcellular molecular arrangements are critical pathological features.

Our ZipAEr model benefits from innovative architectural design and a novel training approach. One of its key innovations is the *compressive* convolutional architecture, which simultaneously reduces dimensionality across both spatial and gene-channel axes. Unlike conventional autoencoders for natural images that typically expand channel depth while reducing spatial size, ZipAEr progressively compresses the 6,000+ gene channels into a compact latent representation. This important compressive behavior of ZipAEr stems from our innovative architectural design tailored to spatial transcriptomics, where gene counts exceed typical imaging channels by three orders of magnitude. In particular, the successful training of our models (ZipAEr2 and ZipAEr3) demonstrates that these high-dimensional data can be effectively encoded in a much smaller latent space, reducing memory requirements by factors of 19 and 163, respectively, which would make complex downstream analyses computationally feasible.

Our results highlight a crucial trade-off between the degree of compression and the fidelity of data reconstruction. The shallower ZipAEr2 model, with its larger latent space, provided a more accurate reconstruction of transcript expression patterns, making it an excellent candidate for detailed downstream analyses where precision is paramount. In contrast, the deeper ZipAEr3 model achieved a significantly more compact representation at the cost of some reconstruction inaccuracy. This makes ZipAEr3 appropriate for exploratory analyses on very large datasets or in scenarios with constrained computational resources. This inherent flexibility allows researchers to select the optimal model architecture based on their specific analytical needs and available hardware.

Another innovation in ZipAEr is the custom *weighted* MSE loss function that we implemented to ensure the model learned meaningful features across the wide dynamic range of gene expression values. Weighting each gene’s MSE by the inverse of its total expression reduced the influence of highly expressed genes, allowing the model to retain signals also from rarer, yet potentially more biologically relevant transcripts.

While we evaluated ZipAEr using CosMx data, it can also be used to analyze data generated by other spatial omics technologies, including 10x Genomics Visium^114^ and Xenium^115^, MERFISH^116^, CellScape^117^, Slide-seq^118^, DBiT-seq^8^, CODEX^9^, HDST^119^, Stereo-seq^120^, sci-Space^121^, Pixel-seq^122^, Seq-Scope^123^, osmFISH^124^, seqFISH^125^ and seqFISH+^126^, ExFISH^127^, EASI-FISH^128^, etc. These technologies differ in spatial resolution and the number of genes and proteins they can measure simultaneously. Consequently, the optimal ZipAEr structure may require a different number of layers and other hyperparameter settings. For example, spatial data spanning the whole transcriptome and several thousand proteins can be processed with a reduction parameter of *r* = 3, which lowers patch tensor memory requirements by a factor of 2.25 compared to *r* = 2. With a batch size of 1 and proper gradient accumulation, ZipAEr can handle datasets with 4.5 times more channels (i.e., genes and proteins) than described in this paper. Furthermore, ZipAEr may have applications beyond spatial omics in scenarios where the input tensor has hundreds or thousands of channels, such as: a) functional brain imaging^129,130^, which involves hundreds of time points or frequency bands per voxel; b) long-term, high-resolution microscopy^131^, which tracks multiple fluorescent markers over hundreds of time frames and channels; c) hyperspectral imaging^132–134^, which captures information across a broad range of the electromagnetic spectrum.

The development of ZipAEr was not without significant computational hurdles. The large volume of the CosMx data necessitated a sophisticated and scalable data processing pipeline. We addressed these challenges by leveraging technologies such as Apache Parquet and Spark for efficient data loading and preprocessing. For model training, we employed a data parallelization strategy with gradient accumulation across a multi-GPU cluster, managed by PyTorch Lightning. This integrated approach made it feasible to train a deep learning model^135^ on spatial transcriptomics data at the terabyte scale.

While ZipAEr represents a significant step forward, it is important to acknowledge its limitations. First, as with any autoencoder, the compression is inherently lossy. The performance decline observed from ZipAEr2 to ZipAEr3 indicates a limit to how much the spatial transcriptomic information can be compressed before critical details are lost. Second, while effective at capturing the local microenvironment, our patch-based approach may not fully encapsulate long-range cellular interactions that extend beyond the predefined patch boundaries. A solution might be to stitch latent representations of neighboring patches together to emulate a super patch. Third, while the model may have implicitly leveraged transcript localization within a cell as an important feature, explicit extraction of this valuable information still requires further work. Fourth, while a gradual change in image size and channel count is generally preferred^136^, we had to reduce the number of channels from over 6,000 in the input layer to 512 in the first layer due to GPU memory constraints. We expect this challenge to be mitigated by technological advancements and a reduced resolution, where appropriate based on the biological context.

Future work can focus on leveraging the rich latent representations generated by ZipAEr for specific biological inquiries, which include developing supervised models for cell-type classification directly from the latent space that could uncover how the microenvironment influences cell identity. We also plan to explore the application of ZipAEr to other high-resolution spatial omics technologies and diverse tissue types to validate its generalizability. Architectural improvements may include alternative convolutional kernel sizes^137,138^ (beyond 3 × 3), integrating multiscale blocks^139^ and residual connections^140,141^, more aggressive resolution reduction, and removing sparse or low-quality patches. Finally, integrating graph neural networks^142–144^ with the patch-based latent representations could provide a powerful framework for simultaneously modeling both local and long-range interactions.

## Conclusion

ZipAEr provides a robust, scalable, and innovative solution for analyzing high-dimensional spatial transcriptomics data. By avoiding cell-level aggregation and instead learning a compressed representation of the local transcriptomic landscape, our method enables a finer exploration of cellular states and microenvironment interactions. ZipAEr is a foundational tool that paves the way for discoveries in the complex and rapidly evolving field of spatial biology.

## Figures and Tables

**Table 1.** Computational resources needed to train ZipAEr models.

Model	Number of parameters	Latent dimensions	Memory usage (GB per GPU)	Training time		Validation loss
				1 sample	100 epochs	

ZipAEr2	61M	512 × 74 × 74	35	23 ms	83 hours	6.1
ZipAEr3	63M	256 × 36 × 36	36	23 ms	83 hours	8.9

## Highlights

1. Compressive convolutional autoencoders can effectively reduce the size of spatial omics data by 2–3 orders of magnitude with minimal loss of biological information.
2. The resulting latent variables are valuable for downstream analyses using machine learning models and for testing biological hypotheses.
